# Translation, adaptation, and validation of the Care Coordination Instrument for cancer patients

**DOI:** 10.1186/s12913-024-12123-4

**Published:** 2025-01-03

**Authors:** Anne Werner, Anke Steckelberg, Alexandra Strobel, Andreas Wienke, Heike Schmidt, Dirk Vordermark, Patrick Michl, C. Benedikt Westphalen, Julia Lühnen

**Affiliations:** 1 Institute for Health and Nursing Science, Faculty of Medicine, Martin Luther University Halle-Wittenberg, Halle (Saale), Germany; 2 Institute of Medical Epidemiology, Martin Luther University Halle Wittenberg, Biostatistics, and Informatics, Halle (Saale), Germany; 3 Department of Radiation Oncology, University Hospital of the Martin Luther University Halle-Wittenberg, Halle (Saale), Germany; 4 Department of Internal Medicine IV, Heidelberg University Hospital, Heidelberg, Germany; 5 Department of Medicine III and Comprehensive Cancer Center Munich, University Hospital, Ludwig-Maximilians University Munich, Munich, Germany; 6 Charité – Universitätsmedizin Berlin, corporate member of Freie Universität Berlin and Humboldt-Universität zu Berlin, Institute of Clinical Nursing Science, Berlin, Germany

**Keywords:** Care coordination, Cancer, Validation, Psychometric evaluation, Questionnaire, Translation, Adaption

## Abstract

**Background:**

Cancer requires interdisciplinary intersectoral care. The *Care Coordination Instrument* (CCI) captures patients’ perspectives on cancer care coordination. We aimed to translate, adapt, and validate the CCI for Germany (CCI German version).

**Methods:**

The original English version contains 29 items in three domains, measured on a 4-point Likert scale (strongly disagree to strongly agree). Validation was conducted in three phases (mixed methods): (I) translation; (II) adaptation: pilot testing and revision in an iterative process using semi-structured, cognitive interviews with patients and professionals (physicians specializing in cancer), with interviews transcribed and qualitatively analyzed by inductive coding; and (III) validation: quantitative validation performed online (LimeSurvey), of at least 80 German patients, each with common cancer (breast, prostate) and rare cancer (different entities), with examination of factor structure (factor analysis) and determination of internal consistency (Cronbach's α) as well as potential influencing factors such as gender, education, or migration background (multivariable regression).

**Results:**

Six patients and six professionals tested the translated instrument for comprehensibility, readability, and acceptability. Two items were consistently problematic for interviewees. A 31-item version (29 items + 2 alternative items) was validated in 192 patients. The alternative items had a higher variance in response behavior and were better understood; therefore, they replaced the two problematic items. However, the three original domains could not be confirmed statistically. Exploratively, a two-factorial structure (with cross-loadings) emerged, which can be interpreted as “communication/information” (16 items) and “need-based navigation” (17 items). Overall, the instrument had a high internal consistency (total score α = 0.931, *M* = 47.16, *SD* = 14.25; communication/information α = 0.924, *M* = 30.14, *SD* = 8.93; need-based navigation α = 0.868, *M* = 23.99, *SD* = 8.37). Significant factors on the care coordination score are treatment location (hospital vs. private practice oncologist *M* = -9.83 score points, *p* = 0.011) and gender (women vs. men *M* = 8.92 score points, *p* = 0.002).

**Conclusion:**

The CCI German version is a valid instrument for measuring patients’ perceptions of cancer care coordination. Both domains reflect important aspects of care. The sensitivity of the CCI should be examined in future studies involving different cancer entities.

**Supplementary Information:**

The online version contains supplementary material available at 10.1186/s12913-024-12123-4.

## Introduction

In a complex healthcare system with a high degree of specialized care, reliable coordination among all partners involved in the treatment process is needed [1]. Coordination is a basic component of care, especially for patients with chronic conditions or diseases that require complex treatment structures, such as cancer, involving multidisciplinary care, extended periods of time, multiple settings, and interventions [2, 3].

In an active treatment situation, multiple factors must be coordinated; comorbidities must be considered; and oncology specialist, primary care provider, and patient participation must be integrated [4]. Comorbidities, in particular, make this task more difficult [4–6]. Additionally, the frequency or rarity of an underlying disease influences the experience of care coordination [7–10]. Therefore, patients with rare conditions perceive less professional support in care coordination and higher personal, familiar, and time burdens when coordinating their own care [8, 10, 11]. Care coordination for rare diseases may be more complex than that for common diseases. For example, there are often fewer choices where they can be treated and a higher number of involved professionals [8]. However, the definition and dimensions of care coordination remain unclear [4, 12, 13]. Care coordination impacts medical (e.g., mortality), system-centered (e.g., costs), and patient-experienced outcomes (e.g., experiences with care) [8, 14–16]. Insufficient care coordination can lead to delays in medical decision-making and mismanagement (e.g., uncoordinated appointments) [8]. Good care coordination is associated with better health status of cancer survivors [17]. Patients who experienced higher continuity of cancer care showed a lower need for supportive care [18]. Medical care coordination is an integral part of process quality and is thus one of the foundations of high-quality medical care [19–22]. Well-coordinated healthcare includes different coordination components such as identification and assessment of the need for coordination services, care planning, and communication from different perspectives (patient, provider and system) [6, 13, 23]. Although theoretical and practical interest in coordinated care has increased [5, 13, 23], research on cancer patients’ perspectives on care coordination is comparatively low [5, 6]. The coordination of cancer care is often viewed from a professional perspective [24–26].

There are care coordination tools in the English language that capture the patients’ perspectives. A questionnaire developed in 2005 (Adapted Picker Institute Cancer Survey) by Ayanian et al. includes eight items on care coordination in a 34-item quality-of-care questionnaire [27]. In 2011, Young et al. developed a care coordination measurement for cancer patients concerning patients’ perspectives on the Australian healthcare system (CCCP-Q) [3]. They found a 2-factor structure with the domain’s “communication” and “navigation.” In 2019, Evensens et al. developed the Consumer Assessment of Healthcare Providers and Systems (CAHPS) for cancer care among all treatment settings and modalities [28]. This questionnaire consists of three care coordination items within a 53-item consumer assessment [28].

Because of the differences in cancer care between Australian and US healthcare systems and some limitations in other measures, Okado et al. (2020b) developed a physician-centered care coordination instrument (CCI) for adult cancer patients in the US [6]. Based on a literature review, the items formulated by Okado et al. (2020b) were tested by oncological nurses and cancer patients, and in the second step, by seven focus groups [6]. Statistical validation was then performed [6]. This instrument includes 29 items on a 4-point Likert scale ranging from *Strongly Agree* to *Strongly Disagree* and three domains: communication, navigation, and operational. The CCI has high reliability with a Cronbach’s α of 0.922.

The German health care system is highly fragmented [29]. Therefore, care coordination is an important factor in ensuring continuity of care and lowering patient burden. Currently, no instruments are available to assess care coordination or capture changes over time. There are three coordination measurements, or measurements with a coordination section, in the German language. None of them focuses on cancer patients’ perspectives, and none of them focuses on the entire treatment process [30]. We decided to translate, adapt, and validate the CCI instrument because of its focus on physicians’ roles in the care coordination process. Despite the differences in the American healthcare system, the focus on the role of physicians is well reflected in the German healthcare system with its physician-centered approach [31].

In order to capture the patients’ view of the care coordination, we are aiming to achieve the following objectives with our study:

1. Translation of the CCI
2. Adaption of the CCI for the German healthcare system via qualitative pilot testing
3. Validation and statistical testing of different predictors

### Notes on reporting

We followed a multistep process comprising the translation, adaptation, and validation of the instrument [32, 33]. We used the SRQR checklist to report the qualitative part of this study [34] and the CROSS checklist to report the quantitative part [35]. Additionally, the reporting recommendations of Streiner and Kottner were considered [36].

In the following, we differ from the classic structure of background, methods, and results, as the methods of a later project phase are based on the results of the previous phase. We believe that reporting the research steps in the correct chronological order helps to comprehensively report the course of the project.

## Phase 1 – translation

### Methods

We used a multistep process to create a cross-cultural instrument [32, 33]. Initially, the CCI was translated into German by a master’s Health Science student familiar with healthcare terminology. After a consensual revision (female master’s student and JL), the instrument was translated back by a native English speaker (female) who was not familiar with the original version [32]. In the next step, the project team (AS, JL, AW) discussed both versions with regard to ambiguities, content-related continuity, and correct terminology, paying attention to the equivalence criteria [33]. Furthermore, we considered comprehensibility, readability, acceptability, and gender-sensitive language. In Germany, the use of gender-neutral or gender-appropriate languages is not uniformly regulated. The use of gender-inclusive languages can lead to sentences that are greatly lengthened.

### Results

We identified the term “cancer doctor” that needed special attention. In a very close translation, we had to use the word “oncologist.” However, not every cancer in Germany is managed by an oncologist. It is also possible for a family or resident doctor to lead the therapy. In addition, the term “oncologist” is a technical term that might be difficult for patients to understand. Therefore, we have chosen the term “betreuender Arzt” (attending physician). We also applied a gender-sensitive language, using neutral phrases or mentioning male and female forms (“betreuender Arzt bzw. betreuende Ärztin”). Inverted items and bipolar response scales were retained. Only the direction of the answer scale changed [37]. For German users, the original direction can be counterintuitive, and a direction from left (disagree) to right (agree) is recommended [37]. We found consensus-based translations for all 29 items.

## Phase 2 – adaptation

### Methods

In February 2022 we recruited participants via snowball sampling and contacted them via email. The physicians recruited were known to the coauthors. The inclusion criterion was work experience with oncology patients. The patients were recruited via self-help groups. The inclusion criteria were personal cancer experiences, aged 18 + , ability to read the questionnaire and the experiences in the German healthcare system. The interviews were conducted in German by AW (female, BSc. psychology, master’s student of psychology), a research assistant at Martin Luther University Halle-Wittenberg. Some respondents were already familiar with interviewer AW or members of the research team, JL (female, PhD) and AS (female, professor), prior to the interview. All persons addressed participated in the interviews. We planned to conduct as many interviews as possible until no new feedback was received. None of the interviews were repeated. Only the interviewer and interviewees were involved during the interviews. Interviews were conducted online via Webex in two iterative cycles in February 2022. We followed both the reparative and descriptive approaches [38]. The interviews began with an introduction to the project and general questions focusing on expectations of care coordination (Supplement 1). Subsequently, the interviewees examined the instruments. They were invited to think aloud. The interviewer (AW) also used verbal probes to examine specific wording. During the revision process, the authors of the original instrument agreed upon uncertainties regarding specific items. All participants were pseudonymized.

Interviews were recorded and transcribed. The transcripts were not returned to interviewees for correction. Field notes were also taken. A qualitative content analysis was conducted with Microsoft Excel. Owing to the limited scope of adapting the questionnaire, we did not make any assumptions about possible changes or difficulties that respondents might have with the instrument. Based on this decision, we used inductive coding to structure feedback and revise the instrument. The revisions were performed iteratively. First, feedback from the first interview cycle was incorporated. Comments on each item were gathered and reviewed along with the respective items. The identified aspects were discussed, and the items were reformulated by consensus between AW, AS, and JL. The reformulated items were tested during the second interview cycle. Comments on the respective items were collected in the second cycle and considered together with the items after completion of the interview cycle.

The interviewer coded all interviews (AW). A secondary coder (JL) checked at least 30% of the coded material. Categories and subcategories were created by consensus between AW and JL. The first coder (AW) had little experience with qualitative research, whereas the second coder (JL) had considerable experience. To illustrate the quotations used in our study, they were translated by AW and retranslated by a native English speaker. Some interviewees asked for feedback after the pilot testing of the instrument was closed and received it via email.

### Results

In the first interview cycle, four patients (three with breast cancer and one with prostate cancer) and three physicians specializing in cancer (internist, radiation therapist, and researcher) participated. In cycle 2, two patients (one with breast cancer and one with prostate cancer) and three oncologists participated (Table 1). The average interview duration was 42 min.

**Table 1:** Participants in the interviews by interview cycle

	Cycle 1	Cycle 2	total
	*n* = 7	*n* = 5	*n* = 12
*profession*			
patient	4	2	6
physician	3	3	6
*sex*			
female	4	1	5
male	3	4	7

Using qualitative content analysis for the statements, 5 categories with 19 subcategories were derived: 1) what constitutes care coordination, 2) transferability, 3) assumptions about patients’ perspectives, 4) linguistic barriers, and 5) content (Table 2). Category one refers to the general understanding and aspects of the “care coordination” construct to be measured, while categories two to five refer to the questionnaire itself. All the categories referred to more than one item.

**Table 2:** Response categories and subcategories of comments

1) What constitutes care coordination	2) Transferability	3) Assumption about patients’ perspectives	4) Linguistic barriers	5) Content
Structure	Differences in healthcare system	Comprehensibility of items	Grammar and sentence complexity	Lack of content related notes
Communication/ information	Translation	Knowledge and understanding	Negations / reverse-coded items	Content duplication
Actors involved	Theory to medical practice		Ambiguity of terms	(individual) Relevance of topics
	Financial issues		Gender appropriate language	Scope of interpretation
			Formal aspects, text structure, and order	Uncertainty due to the question

### What constitutes care coordination

This category includes statements about what constitutes care coordination, who is involved, and which aspects are part of care coordination. We have elaborated on this point to ensure that the concept of care coordination underlying the instrument is understood similarly by the participants.

(PH = Physician, P = Patient).

Structure: Patients and physicians reported the need for structured treatment and fixed contacts as part of good care coordination. Physicians also reported that good coordination is based on patient preferences.

PH6 (not item related): “Good coordination is based on patient preferences and enables straightforward access to diagnostics and therapy.”

P6: “So, the first thing that comes to my mind is consistent personal contact.”

Communication/information: This category contains statements on the necessity of regular communication between all individuals involved in the treatment, including patients, who must be able to understand which treatment step happens, where, and why. One patient expressed the need for personal access (how and when) to the attending physician.

PH3 (not item related): “[…] education about various other options such as rehabilitation, social-support groups et cetera.”

P6 (not item related): “... So, a certain form of personal availability, even if it is only by email. So [...] I would consider that as being quite flexible. And actually, easy accessibility. That is something I’ve often become aware of now. In other words, clarity about when people can be reached and how, in what format is best? So, whether by phone or by email. How quickly can you expect an answer? In order to make sense of something like appointment coordination.”

Actors involved: Patients and physicians reported the importance of interdisciplinary and intersectoral professional cooperation and decision-making in the treatment process which professions (medical and psychological) and which non-professional, non-medical stakeholders should be involved in coordination (e.g., support groups, cancer societies, and apps).

PH4 (not item related): “A good care structure also means that a specialist no longer decides alone how the treatment of a tumor patient is to be carried out, but that it must happen in a tumor board nowadays, where the various disciplines are always brought in from one stage to the next, depending on where one is at the time. And by specialists, I mean not only the various medical disciplines, but also the nursing disciplines. That palliative medical care is also involved in good time.”

P3 (not item related): “Good coordination would have been [...] if the disciplines would talk to each other more often from time to time. Towards the end of the whole thing, the obstetricians asked: What did the oncologists say, how much break do we need? And I said: Well, I do not know, I thought they were talking to you.”

### Transferability

This category includes various barriers to cross-cultural and cross-linguistic transfers. Patients and physicians expressed concerns about the transferability of a US care coordination instrument to the German healthcare system. This highlighted specific concerns that arose when translating the tool into German.

Differences in healthcare system: Interviewees (only physicians) doubted the general possibility of transferring the instrument to the German health system because of differences between the health systems. They reported their experiences with the US healthcare system. In particular, they mentioned the various responsibilities of for example different professional groups and outpatient and inpatient sector in the German healthcare system. Quotes for this category were formulated only in the first interview round. After the first adaptation, in the second interview cycle, these aspects were not mentioned.

PH5 (feedback for the entire questionnaire): “You’re stirring up a hornet’s nest specific to Germany. Because in the USA, the gynecologist operates. If its cancer, the patient goes to the oncologist. Or the dermatologist operates. Its cancer, he goes to the oncologist. In Germany, the gynecologist operates, then does the chemotherapy himself and so on. So, we have this organ oncology. And the German oncologists don’t like it very much, because everyone does a bit of chemo.”

Translation: Participants reported difficulties with certain translated terms and phrases. For example, the English word “test” is not equivalent with the German word “Test.” Therefore, a different term must be identified.

PH4 (item 4): “Yes, question four seems to me also again, because you took it from English, the German patient speaks of “Untersuchung” (=examination). He goes to an “Untersuchung” and not to a test.”

Theory to medical practice: Patients and physicians reported situations and regular procedures that differ from the facts and circumstances. This is the most frequently mentioned criticism. In particular, items no. 5 and 17 (see Supplement 2) have been repeatedly criticized for their differences in treatment practice.

PH6 (replaced item): “[...] you’ll get a 100% “don’t agree at all” answer, I’m sure. So that’s a hard question. [...] something like that happens in the very rarest [cases], [...] where there are [...] Breast Cancer Nurses [one could imagine] that they do that or something. But that someone from the treatment team, especially in the case of outpatients, would ask: How is it going? No.”

Financial issues: The interviewees were asked about their understanding of the financial aspects of healthcare. One item in particular (“I was informed of financial aspects of cancer care.”) drew a connection between the financial burdens of cancer treatments. Owing to the insurance situation in Germany, treatment itself is not a burden for patients. Nevertheless, cancer patients experience a financial burden owing to reduced income during sick leave. Despite the fact that treatment costs are generally covered by health insurance, patients must pay a co-payment for treatment or pay in advance. The interviewed participants noted that it was not the treatment that led to financial burdens, but the disease. They mostly understood the term in the same way and named similar financial burdens that patients face due to cancer illness.

PH2 (item 9): “Well, costs for examinations and therapies that patients may have to bear themselves. But also, the issue of loss of earnings due to long periods of illness.”

### Assumption about patient’s perspective

This category contains all the assumptions that the respondent makes about effects on (other) patients. This does not imply the difficulties and criticisms that the respondents themselves faced with the instrument.

Comprehensibility of items: Patients and physicians assumed that (other) patients would not understand an expression or term or would not understand the whole item because it was too complex or ambiguous.

PH4 (item 8): “So, this sentence has such a complicated structure that most patients would already, I think, stop thinking about it.”

Knowledge and understanding: This category contains all statements that concern the assumption that (other) patients cannot understand the questioned facts because of missing information or the impossibility of knowing certain facts. The interviewees assumed that patients would not answer truthfully, would have a fixed response tendency, the response behavior would be determined by general social prejudices, or the patient would not have access to the “true” answer.

P4 (item 15): “The patient can hardly assess whether the doctor has done all the necessary diagnostics.”

PH4 (item 10): “I have a comprehensive understanding about my treatment plan. So even I as a professional colleague wouldn’t always want to answer that so simply. Because I just read again […], the more informed you are, the more you know your limits.”

### Linguistic barriers

This category contains all the interviewees’ statements on linguistic barriers, such as grammatical problems, sentence complexity, negations, ambiguity of terms, and gender-appropriate language.

Grammar and sentence complexity: Patients and physicians expressed irritation due to tense, subjunctive/indicative formulations, or active/passive formulations, and ambiguity due to too high sentence complexity.

P4 (item 8): “Yes. I think that in the case that I/ Oh, very long sentence and very complicated.”

Negations/reverse-coded items: Physicians expressed a lack of clarity due to negated statements in the reverse-coded items. They preferred a positive formulation of items.

PH2 (item 16): “Yes, the 16 is very striking, it’s out of the ordinary, isn’t it? With this “not”. I don’t know, either it’s sometimes an oversight in questionnaires, that something strange is put in at one point, which is difficult to evaluate afterwards, or it’s put in on purpose to test whether the patient really reads how it’s formulated. [...] You stumble over it a bit. The questions around it are formulated positively, so/ So if “it completely applies”, then it’s just good, so you have the information, you know, I know where to call and so on. And now “applies completely” would be a negative situation, so to speak. But okay, if that’s how it is in the original design.”

Ambiguity of terms: Physicians and patients criticized meaningful terms and formulations that were unusual, vague, or too specific.

P4 (item 16): “What is important is not “how much” support I need but rather "whether" I need support at all.”

Gender-appropriate language: Patients and physicians expressed irritation due to the gender-appropriate formulation strategy.

PH4 (introduction): “...Furthermore, I understand that your institute is also very committed to gendering, but this constant listing of both genders makes it a bit difficult, especially since it is not guaranteed throughout. And maybe there could be a sentence in front of it. Why don’t you always write the “female doctor,” that’s fine? Yes, but if you limit yourself to one gender and include that in the introductory text, then the questionnaires will be clearer afterwards.”

Formal aspects, text structure, and order: Patients and physicians expressed their wish for another structure of the introductory text, structuring by bullet points, thematic sorting of items, or a changed order within an item.

PH3 (item 11): “Is the alternation between the topics intentional? I would find it better to cluster the questions around the topics - this creates the feeling of duplication.”

### Content-related notes

All feedback regarding the content of the items was included. This refers to statements on the answerability of the items, comprehensibility of the instructions for completing the questionnaire, missing topics, perceived duplication of content, and vague and unspecific item content. Topics that the respondents considered important were also included.

Lack of content-related notes: Patients and physicians would like an additional item to represent a further aspect that has not yet been addressed (e.g., special social services and onco-nurses) or to focus on an aspect more strongly (e.g., psycho-oncological support).

P4 (item 10): “Yes. And you should also ask whether the partner or a relative was invited to the appointment, yes? Because under the shock of the diagnosis, the person affected cannot absorb everything. It all rushes past them. And then it is at least important that a relative is also present.”

Content duplication: Patients and physicians noticed the duplication of content or related constructs in the two items.

P5 (item 24): “Question already similar above.”

(Individual) relevance of topics: The interviewees considered the topic important.

PH4 (item 18): “That’s incredibly important, isn’t it? Because again and again I see colleagues who are so convinced of themselves that they refuse a second opinion and then also say: "Then you don’t need to come back." I’ve seen it all, yes. That’s why it’s very, very important.”

Scope of Interpretation: The interviewees noted that the item content was not clear, not sufficiently specific, and formulated too vaguely or too globally. Three items (no. 2, 3, and 29 in Supplement 2) in particular received this criticism:

PH2 (item 29): “Yes, that’s a bit of a global assessment. I was wondering now, coordinated, so between whom? [...] A bit unspecific. Or probably too global.”

Uncertainty due to the question: Interviewees became insecure because of the question or thought that it could make others feel insecure.

P2 (item 12): “I don’t know if that applies to everyone. But I wouldn’t bring up the number 12 with the patient. Because I think it only gives rise to reservations or people tend to think: OK, is something wrong here? I would think about whether I would ask about that.”

One of the 29 items remained unchanged. The remaining 28 were adjusted. The adjustments concerned, for example, gender-appropriate language, changes in meaningful (but ambiguous) terms, and sentence complexity. While some items received less or moderate critical comments, two received constant criticism or indifferent remarks (no. 17 and 23 in Supplement 2). The main criticism was the mismatch between the facts questioned and the reality of medical practice. However, the patients also noted that they wished to have this experience. Owing to the strong denial on the part of patients and physician that the questioned facts had ever been experienced, we assumed a low variance in the answers to this item in further surveys. Interviewees understood the question in different ways and used different circumstances as the basis for their answers. Therefore, we generated experiential equivalence items that narrowed down the questioned facts or asked for an experience-based alternative to the original questioned facts for these two items [33].

## Phase 3 – validation

### Methods

To validate and statistically examine our CCI adaptations, we conducted a cross-sectional online survey using LimeSurvey from 3/22/2022 to 8/18/2022.

#### Sample, recruitment, and data collection

The target groups were people over 18 years old with rare cancer diseases, regardless of location, and people over 18 years old with the most common cancer diseases in Germany, i.e., breast cancer and prostate cancer [39]. Based on the literature and knowledge of the German healthcare system, we assumed that there are differences in the coordination of cancer care between rare and common cancers. The aim was to recruit a minimum of 80 patients (convenience sample) for each of the rare and common cancer groups. The target value was arbitrarily set based on the feasibility estimate but is also consistent with the item-response-ratio. This ratio recommended a minimum sample in the ratio of the number of items of 3:1 to 6:1 [41, 42].

First, recruitment was conducted by the participating physicians (DV, PM, and CBW). They distributed flyers to the patients. In addition, we used snowball sampling, recruited participants via personal contacts, and sent emails to 102 support groups in nine of the sixteen federal states and to patient organizations throughout Germany. Generally, surveys were conducted online. However, participants could choose to use paper-and-pencil forms (*n* = 5). All the participants were anonymized using consecutive numbering.

#### Questionnaire

The questionnaire began with informed consent, followed by 31 CCI German items (29 original items and 2 alternative items; see Supplement 2). Additionally, there were two follow-up questions, one for the problematic item 23 and one for the corresponding alternative items, to capture the intention of the given answer (supplement 2). In addition to the CCI German version, we collected medical data, including initial diagnosis, comorbidity, treatment location, type of insurance, actual treatment, involvement of an oncological navigation service (“Onkolotse”), and frequency of medical consultations by using 15 multiple-choice questions developed for this study. Furthermore, personal data such as age, sex, education, migrant background, employment status, and living situation were collected.

The survey either consisted of 6 paper pages or 33 online pages. On average, the completion of the questionnaire (CCI and sociodemographic questions) took 11.8 min.

#### Statistics

Descriptive analyses were conducted using IBM SPSS Statistics 28 for medical and sociodemographic data (AW). First, we compared the two problematic CCI German version items with their alternatives and decided which one was to be used further (AS, JL, and AW). We intended to make this decision based on the statistical (variance, discriminatory power, and factor loading) and content-related criteria. Therefore, further analyses of the CCI German version included only 29 items. For the statistical analyses of the CCI German version, we followed the scoring guide of the original CCI (e.g., handling inverted items). Discriminatory power (item-total correlation) was calculated using IBM SPSS Statistics 28. The original instrument had a three-dimensional structure (communication, navigation, operational). To evaluate the structure of the German version, we conducted confirmatory factor analysis using R Studio (AStr). Principal component analysis with varimax rotation was performed. To evaluate possible predictors, such as sex, education, migration background, and medical factors, we assessed a multivariable regression in R Studio. To assess group differences (male vs. female) in age, a t-test was used; for other socioeconomic and medical data, the χ^2^-test was used. Data weighting was not performed.

Based on the results of Okado et al. (2020a) [2] and research on care coordination, we additionally focused on the associations between CCI German version scores and the characteristics sex, migration background, education, time since diagnosis, and comorbidities [2, 4–6, 14, 27, 43].

### Results

#### Participants

Of the 102 support groups contacted, 17 responded with the promise of distributing the survey link. The questionnaire was accessed 292 times. A total of 179 participants completed the CCI German version completely. A total of 192 participants completed at least as many items as needed to build one domain and were included in the calculations. Participants were on average 59.3 years old (*SD* = 12.7, Range 29–85), mainly female (61.1%), had a high education level (higher school/college certificate, 45.0%), and lived in a partnership (83.3%) (Table 3).

**Table 3:** Characteristics of participants

	Female *n* = 116	Male *n* = 74	Group difference female vs. male	Total *n* = 192
**Sociodemographic data**	*n (%)*	*n (%)*		*n (%)*
*Age (n* = *188), mean (SD)*	55.2 (11.6)	65.8 (11.6)	*p* < 0.001	59.3 (12.7)
*Sex (n* = *190)*				
Male	-	74 (100)		74 (38.5)
Female	116 (100)	-		116 (61.1)
*Education (n* = *189)*			*p* = 0.323	
Lower secondary education (9 years)	8 (6.9)	9 (12.3)		17 (9.0)
Middle school (10 years)	33 (28.4)	14 (19.2)		47 (24.9)
Technical college (12 years)	17 (14.7)	14 (19.2)		31 (16.4)
Higher school/college (12 years)	51 (44.0)	34 (46.6)		85 (45.0)
Others	7 (6.0)	2 (2.7)		9 (4.8)
*Employment status (n* = *187)*			*p* < 0.001	
Employed fulltime	17 (14.7)	16 (22.5)		33 (17.6)
Employed part-time	29 (25.0)	1 (1.4)		30 (16.0)
Unemployed	3 (2.6)	0 (0.0)		3 (1.6)
Retirement pension	22 (19.0)	38 (53.5)		60 (32.1)
Civil servants' pension	6 (5.2)	4 (5.6)		10 (5.3)
disability pension	17 (14.7)	9 (12.7)		26 (13.9)
Incapacity for work	11 (9.5)	2 (2.8)		13 (7.0)
Others	11 (9.5)	1 (1.4)		12 (6.4)
*Marital status (n* = *186)*			*p* = 0.002	
Single	27 (23.5)	4 (5.6)		31 (16.7)
In partnership	88 (76.5)	67 (94.4)		155 (83.3)
*Migration Background (n* = *186)*			*p* = 0.435	
Yes	12 (10.4)	5 (7.0)		17 (9.1)
**Medical Data**				
*Treatment location (n* = *181)*			*p* < .001	
Hospital	40 (36.7)	27 (37.5)		67 (37.0)
Private Practice Oncologist	17 (15.6)	13 (18.1)		30 (16.6)
Gynecologist/ Urologist	22 (20.2)	18 (25.0)		40 (22.1)
Family doctor	12 (11.0)	4 (5.6)		16 (8.8)
Others^a^	18 (16.5)	10 (13.9)		28 (15.5)
*Cancer frequency (n* = *190)*			*p* = 0.691	
Common	52 (44.8)	31 (41.9)		84 (43.8)
Rare	64 (55.2)	43 (58.1)		108 (56.3)
*Cancer entity (n* = *192)*			*p* < 0.001	
Breast Cancer	52 (44.8)	0 (0.0)		53 (27.6)
Prostate Cancer	0 (0.0)	31 (41.9)		31 (16.1)
Neuroendocrine tumors	39 (33.6)	38 (51.4)		77 (40.1)
Thyroid cancer	17 (14.7)	4 (5.4)		22 (11.5)
Other	8 (7.0)	1 (1.4)		9 (4,7)
*Chronic condition (n* = *180)*			*p* < 0.001	
Yes	48 (44.0)	49 (69.0)		97 (53.9)
*Using navigation service (“Onkolotse “) (n* = *184)*			*p* = 0.300	
Yes	9 (8.0)	3 (4.2)		12 (6.5)
*Frequency of doctor appointments in the last 3 months (n* = *184)*			*p* = 0.406	
None	6 (5.4)	3 (4.2)		9 (4.9)
1 – 3 times	47 (42.0)	40 (55.6)		87 (47.3)
4 – 6 times	32 (28.6)	16 (22.2)		48 (26.1)
7 – 9 times	13 (11.6)	5 (6.9)		18 (9.8)
10 – 12 times	2 (1.8)	3 (4.2)		5 (2.7)
More than 12 times	12 (10.7)	5 (6.9)		17 (9.2)
*Medical treatment in the last 3 months (n* = *192)*			*p* = 0.333	
Yes	80 (69.0)	46 (62.2)		126 (65.6)
*Insurance status (n* = *189)*			*p* = 0.234	
Statutory insurance	88 (75.9)	60 (82.2)		148 (78.3)
Statutory insurance with private supplementary insurance	12 (10.3)	2 (2.7)		14 (7.4)
Private insurance	13 (11.2)	10 (13.7)		23 (12.2)
Others	3 (2.6)	1 (1.4)		4 (2.1)

^a^Other treatment locations mentioned: breast center (*n* = 2), endocrinology (*n* = 4), radiologist/nuclear medicine (*n* = 4), multiple locations, and others (*n* = 18)

There were significant differences between male and female patients in terms of age, employment status, marital status, cancer type, and chronic conditions. The female participants were younger (*M* = 55.2 years), more frequently employed part-time, less frequently in partnerships, and had fewer chronic conditions.

#### Care coordination-total score

First, we examined two problematic items along with the initial items and their alternatives. The translation of the original item, “Someone from my doctor’s office reaches out and contacts me after visits to check whether I have any problems or concerns,” had a very low mean (0.56, *SD* = 0.71) and median (0). Less than 10% of participants agreed or strongly agreed with this statement. The alternative item, “If something remained unclear, someone from the treatment team will get in contact after my visits to clarify this issue.” had a higher mean (1.10, *SD* = 0.88) and median (1.0). Thirty percent of participants agreed or strongly agreed with this statement. The discriminatory power and variance of these items were either close to each other or low. We decided to use an alternative item instead of the original because of the higher variance in response behavior and, above all, content-related considerations resulting from the interviews. Statistically, this decision improved Cronbach’s α from 0.928 to 0.931.

The second problematic item was “If I had a serious symptom at home, the first thing I do is to go to the emergency room”. This inverted item aimed to evaluate information regarding emergency numbers. Participants who agreed or strongly agreed were asked to indicate their thoughts when they answered. Patients agreed strongly in nearly equal numbers (43.2% to 60%) for all choices: “The symptom is so severe that I can only be helped in the emergency room,” “All other possibilities, for example, calling the emergency number, have already been tried,” “Going to the emergency room was recommended to me by my doctor for such a case,” and “I don’t know who else to contact.” The item was understood very differently and so we decided to use the alternative item “I have received information about which persons/institutions I can turn to if a serious symptom occurs at home.” Most of the participants who agreed or strongly agreed (78%) could name the location they would visit in case of serious symptoms.

Overall, the CCI German version had a high internal consistency with Cronbach’s α = 0.931 (*M* = 47.16, *SD* = 14.25, Range 10 to 87). The total score was normally distributed (Shapiro–Wilk test *p* > 0.05, visual assessment using Q-Q plot). The item-total correlation was between 0.05 and 0.73 (Supplement 2). Only two items had an item-total correlation of less than 0.3. For reasons of content, neither item can be excluded.

#### Care coordination dimensions

**Fig. 1:**
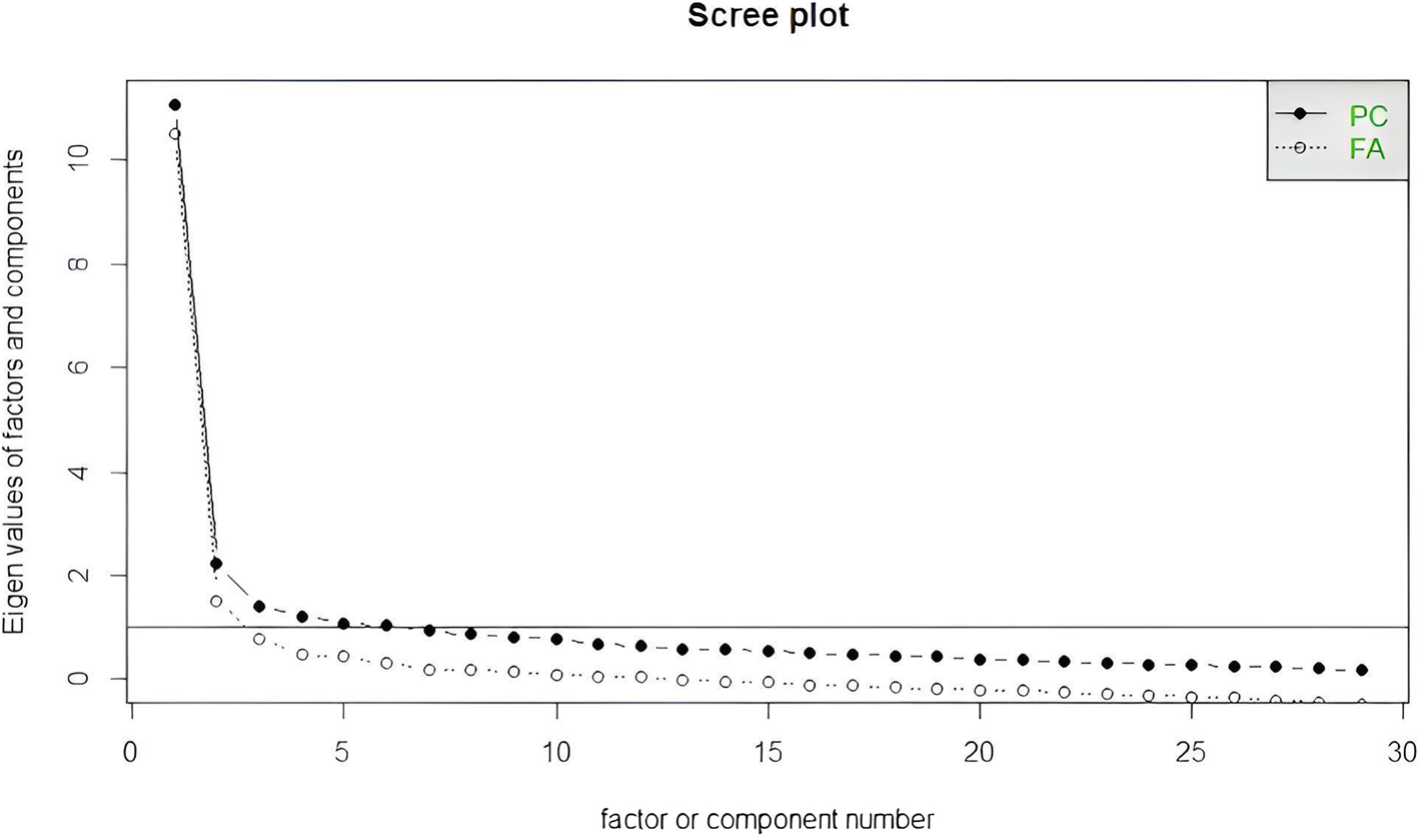
Scree plot with eigenvalue. PC = principal component, FA = factor analysis

The original factor structure with three dimensions could not be confirmed. We used the following indices to measure model fit: Chi-square, Root Mean Square Error of Approximation (RMSEA) and the Standard Root Mean Square Residual (SRMR), Comparative Fit Index (CFI), Tucker Lewis Index (TLI). The confirmatory factor analysis resulted for the three-dimensional structure in a model fit of χ^2^_df=336_ = 592.98, *p* < 0.001, RMSEA = 0.065, SRMR = 0.065, CFI = 0.875, and TLI = 0.859. Therefore, the three-dimensional model missed every benchmark for a good model. Instead, a scree plot and an eigenvalue criterion of > 1 indicated two dimensions (Fig. 1).

Eleven of the 29 items cross-loaded onto both domains. The accepted cut-off for loadings was 0.3. These two items did not load onto any domain. Based on the content and statistics, 25 items were allocated to one of the two domains and four items were related to both domains. The Bartlett test (χ^2^_df=406_ = 2502.51, *p* < 0.001) and the Kaiser–Meyer–Olkin measure of sampling adequacy (KMO = 0.924) confirmed this two-dimensional structure.

Dimension 1 (“communication/information”) includes all processes, communication, and information within the attending physician’s practice (16 Items). The values in this dimension range from 0 to 48.

Dimension 2 (“need-based inter-professional navigation”) describes the need-assessment and need-based navigation outside the caregiver’s institution (17 items). This dimension includes all stakeholders in the healthcare process. Two items that did not load on any dimension were allocated by content to dimension 2. The values in this dimension range from 0 to 51.

For better comparability, both ranges were converted to a scale of 0 to 100 points. Dimension 1 received higher approval ratings than Dimension 2 (Table 4).

**Table 4:** Statistical data for the two dimensions and the total score

		Cronbach’s α	Mean (SD)	Mean (SD), converted scale 0–100
Domain 1: communication/information *(16Items)*		0.924	30.14 (8.93)	62.79 (18.61)
Domain 2: need-based inter-professional navigation (*17 Items*)		0.868	23.99 (8.37)	47.05 (16.41)
Total score (*29 Items)*		0.931	47.16 (14.25)	54.21 (16.38)

Domain 1: maximum range 0–48, Domain 2: maximum range 0–51, Total score: maximum range 0–87

The final CCI German version and the scoring guide can be found in Supplement 3.

Two items did not load onto any domain. In the original version, one of these items (“Mich hat ein Familienmitglied, eine Freundin oder ein Freund unterstützt, meine Krebsbehandlung zu koordinieren.“/ „I have a family member, a close relative or a friend who helped coordinate my cancer care.“) did not load on any domain. The other item (“Manchmal werden bei mir Untersuchungen doppelt durchgeführt.” / “Sometimes I have duplicate tests.“) loaded on the domain „operational.“ In the German version, both items were placed by content in domain 2”need-based inter-professional navigation.”

#### Group differences

Table 5 shows group differences for total score of the CCI German version and for each of the two dimensions.

**Table 5:** Group differences in multivariable regression

	**Dimension 1**		**Dimension 2**		**Total score**	
	**Regression coefficient (95% CI)**	***p*-value**	**Regression coefficient (95% CI)**	***p*-value**	**Regression coefficient (95% CI)**	***p*-value**
*Intercept*	17.54 (−5.66; 40.75)	0.137	23.87 (3.04; 44.69)	0.025	35.16 (−1.20; 71.52)	0.058
*Sex (Ref: female)*						
Male	4.06 (0.44; 7.68)	0.003	5.78 (2.56; 9.00)	< 0.001	8.32 (2.59; 14.05)	0.005
* Age*	0.003 (−0.01; 0.01)	0.524	0.0007 (−0.01; 0.01)	0.871	0.003 (−0.01; 0.02)	0.706
*Education (Ref: 9 years of lower secondary education)*						
Middle school (10 years)	−2.81 (−8.63; 3.01)	0.342	−3.20 (−8.27; 1.88)	0.215	−6.08 (−15.25; 3.09)	0.192
Technical college (12 years)	−3.11 (−9.05; 2.84)	0.303	−4.54 (−9.80; 0.72)	0.090	−6.05 (−15.48; 3.37)	0.206
Higher Schools/ College certificate (12 years)	−3.02 (−8.34; 2.31)	0.264	−3.84 (−8.54; 0.86)	0.109	−6.60 (−14.99; 1.80)	0.122
Others	−4.74 (−13.40; 3.92)	0.281	−4.31 (−12.03; 3.40)	0.271	−7.97 (−21.55; 5.61)	0.248
*Marital status (Ref: Single)*						
In partnership	3.07 (−1.04; 7.19)	0.142	−0.10 (−3.72; 3.53)	0.959	2.95 (−3.50; 9.39)	0.367
*Migration Background (Ref: no)*						
Yes	−0.65 (−5.72; 4.43)	0.801	1.08 (−3.74; 5.90)	0.801	2.36 (−6.04; 10.76)	0.580
*Cancer frequency (Ref: common)*						
Rare	−0.08 (−4.11; 3.94)	0.967	−2.74 (−6.29; 0.81)	0.129	−2.90 (−9.31; 3.52)	0.373
*Treatment location (Ref: Hospital)*						
Private Practice Oncologist	−4.08 (−8.83; 0.66)	0.091	−6.38 (−10.58; −2.18)	0.003	−10.61 (−18.13; −3.08)	0.007
Gynecologist	2.03 (−3.90; 7.96)	0.499	1.19 (−4.17; 6.54)	0.662	2.41 (−6.93; 11.74)	0.611
Urologist	−6.41 (−13.10; 0.27)	0.060	−7.25 (−13.46; −1.04)	0.022	−10.89 (−21.97; 0.18)	0.054
Family doctor	−4.05 (−10.36; 2.25)	0.206	−4.57 (−10.13; 0.99)	0.107	−8.66 (−18.56; 1.23)	0.086
Others	0.33 (−3.88; 4.54)	0.876	−0.85 (−4.64; 2.94)	0.657	−0.46 (−7.10; 6.19)	0.892
*Using navigation service (“Onkolotse “) (Ref: yes)*						
No	0.52 (−5.81; 6.87)	0.870	−3.85 (−9.35; 1.65)	0.169	1.66 (−11.60; 8.28)	0.742
*Chronic condition (Ref: yes)*						
No	0.73 (−2.35; 3.81)	0.641	0.74 (−2.01; 3.50)	0.641	0.62 ( −4.28; 5.53)	0.802
*Medical treatment in the last 3 month (Ref: yes)*						
No	3.12 (−0.35; 6.59)	0.078	2.15 (−1.00; 5.30)	0.078	4.23 (−1.32; 9.77)	0.134
*Frequency of doctor appointment (Ref: none)*						
1 – 3 times	3.81 (−2.92; 10.53)	0.265	3.45( −2.64; 9.54)	0.265	6.56 (−4.07;17.18)	0.224
4 – 6 times	2.97 (−4.00; 9.94)	0.402	3.48 (−2.83; 9.79)	0.278	6.40 (−4.58; 17.38)	0.251
7 – 9 times	3.58 (−4.54; 11.70)	0.384	1.43 (−5.76; 8.62)	0.695	3.94 (−8.80; 16.68)	0.542
10 – 12 times	−1.13 (−11.60; 9.33)	0.831	−3.23 (−12.71; 6.26)	0.502	−3.91 (−20.36; 12.53)	0.639
More than 12 times	5.61 (−2.60; 13.83)	0.179	5.94 (−1.51; 13.39)	0.117	10.03 (−2.91; 22.97)	0.128
*Insurance status (Ref: statutory health insurance)*						
Statutory health insurance with private supplementary insurance	1.62 (−3.94; 7.18)	0.565	3.56 (−1.50; 8.62)	0.167	4.15 (−4.68; 12.98)	0.355
Private health insurance	3.97 (−0.79; 8.72)	0.101	3.84 (−0.41; 8.09)	0.076	6.94 (−0.52; 14.42)	0.069
Others	−0.39 (−10.17; 9.40)	0.937	2.55 (−6.28; 11.38)	0.569	2.82 (−12.53; 18.17)	0.712
* Time since first diagnosis in months*	0.19 (−0.07; 0.45)	0.151	0.21 (−0.02; 0.45)	0.075	0.32 (−0.09; 0.74)	0.126

A strong predictor for group differences in dimension 2 (“need-based inter-professional navigation”) and the total score is the treatment location. Patients treated by a private practice oncologist scored on average 10 points lower than patients treated in a hospital (*p* = 0.007). Patients treated by a urologist had up to 7 points less in dimension 2 (“need-based inter-professional navigation”) than patients treated in a hospital (*p* = 0.022). This is remarkable because male patients, the only patients who were treated by a urologist, had a higher average score than female patients. Patients treated by a family doctor had lower scores than those treated at a hospital.

Sex was a strong predictor of group differences in all the dimensions. Male patients scored 8 points (total score) higher than female patients (*p* = 0.005). The presence of a migration background was a weak predictor (maximum of 2 score points). Different levels of education showed noticeable differences in scores. Therefore, patients with higher education levels had, on average, fewer points than those with a lower secondary education (9 years). The weakest predictors for any group difference were the presence of a chronic condition and the time since the first diagnosis.

Patients with more than 12 doctor’s appointments per quarter scored up to 10 points higher than those with no appointments.

The predictor health insurance showed a noticeable effect of a maximum score of 6 points on the total score for patients with private health insurance compared to patients with statutory health insurance. Examining the item “I felt like my care was impacted by the type of insurance I have" also showed no strong influence of the insurance status on the item behavior.

A comparison of the groups’ male and female participants at the item level is shown in Supplement 4. In general, males had higher mean scores than females. Only in three items did females have higher mean scores than males. However, these differences were not statistically significant.

## Discussion

The translated and adapted German version of the CCI is a valid instrument for measuring patient perceptions of care coordination.

The translated questionnaire demonstrated excellent internal consistency, with a Cronbach’s alpha of 0.931 for the total score, suggesting that the items reliably measured the intended construct in the new language. This high reliability indicates that the translation process was effective in preserving the meaning and structure of the original items. The internal consistency of the CCI German version total score and “communication” domain is similar high to the internal consistency of the original CCI (Cronbach’s α = 0.922) [5, 6]. However, while the internal consistency of the “navigation” domain in the original CCI is only acceptable (Cronbach’s α = 0.793), the corresponding domain “needs-based navigation” in our instrument can be rated as good with a Cronbach’s α of 0.868. We were able to replicate the results of the original. Therefore, we concluded that we could reliably measure care coordination and its two domains of “communication” and “need-based navigation” using the CCI German version. It is not surprising that we achieve a high Cronbach’s α, as the US original also achieves high Cronbach’s alpha values [6]. However, Cronbach’s α has been noted to be influenced by the number of similar items, which may suggest some degree of redundancy when values are exceptionally high [44].

The cognitive interviews led to changes in nearly every item (e.g., simpler formulations and changes to increase sentence comprehension). Two problematic items can be identified and replaced with alternative experience-based items.

Many participants’ expectations of care coordination were reflected in the CCI German version. The instrument addressed the participants’ perceptions of communication/information, treatment structure, and involved actors. Cognitive interviews improved 28 of the 29 items.

The two expected cultural adaptations concerned the financial aspects of cancer care and impact of health insurance. In the US health system, financial barriers to treatment play a greater role than in the German health system. In the US, approximately 10% of the population under the age of 65 years has no health insurance, whereas in Germany, less than 0.1% of the population is without health insurance [27, 45, 46]. Surprisingly, the items in question were well understood by the participants and were adapted to the German health system. Therefore, in Germany the treatment is not the main financial burden for cancer patients, but the illness itself, which leads to the fact that there are a lot of financial burdens following the disease. Both patients and physicians named similar financial burdens that patients face due to cancer illness. Most items were criticized regarding the wording (ambiguity terms). In addition, terms that showed a mismatch between the questioned facts and the medical practice were criticized.

Both patients and doctors made assumptions about the ability of (other) patients to understand the items (“Assumption about patients’ perspectives”). However, patients themselves did not report any difficulties in understanding the difficulties attributed to them This could be explained by the bias of the physicians (stereotypes) or the non-representativeness of the interviewed patients.

The replaced item (“Someone from my doctor’s office reaches out and contacts me after visits to check whether I have any problems or concerns”) that no patient or physician has ever experienced but was stated as a wish by some patients was replaced with an equivalent item that covers the fact but does not have this strong direction. Therefore, we do not think that we have removed an important fact for patients from the questionnaire but that we can continue to cover this fact with a less strict equivalent.

Of the 102 support groups, only 17 responded with the promise of sharing their questionnaires. However, due to the large number of requests, enquiries are not always passed on to group members. In addition, we closed the survey once the sample was complete. Maybe that excluded other members of the self-help groups from our study. Our target sample was achieved and we arrived at an item-response ratio of around 1:6, like recommended Rummel (1988), Hatcher (2014) and Catell (1987) [40–42]. We acknowledge that some literature suggests higher ratios, such as 1:10 or even 1:30 [47–49]. However, there is a lack of empirical evidence to support these recommendations [48]. However, as these larger sample sizes are undoubtedly desirable, we recommend conducting future studies with the questionnaire with larger samples.

The analysis of the questionnaire identified two dimensions ("communication/information" and "need-based inter-professional navigation") each reflecting key aspects of the healthcare process.

These two structures were also observed in CCCP-Q by Young et al. (2011) [3]. The CCCP-Q also includes communication and navigation domains. In an expert panel, Benito et al. (2018) identified two quality indicators of care coordination: management coordination (referral criteria and prompt referral) and information coordination [1]. The US original, on the other hand, consisted of three dimensions, two of which (communication and navigation) were reflected in the structure. The third and unique dimension of the US CCI, “operational” asses the efficiency of care and could not be found in our data. This may be related to cultural differences, and the items from dimension 3 are more communicative or navigational domains than efficiency in Germany.

As in the original US CCI, the item related to support from family and friends did not load onto any domain. This construct appears to be a special aspect of care coordination and should be investigated further. Overall, the two domains (navigation and information) appeared to be fundamental and highly significant.

Compared to the results of Okado et al. (2020a) and Okado et al. (2020c), the total score for patient-perceived care coordination was lower in our study (*M* = 59.6, *SE* = 1.4, *M* = 59.4, *SD* = 11.2; vs. *M* = 47.16, *SD* = 14.25) [2, 50]. However, due to the lack of cut-off values, no assessment of the quality-of-care coordination can be made. Further studies should address the assessment of the quality of scores achieved by the CCI German version.

Associations between migration background, chronic conditions, and time since the first diagnosis in connection with care coordination could not be confirmed. Different levels of education showed a non-significant but noticeable difference in scores. This was particularly noticeable because patients with higher education levels, on average, scored fewer points. This correlation was already evident in the studies by Ayanian et al. (2005) on patients with colorectal cancer, Mora-Pinzon (2019) in a survey on breast cancer patients, and Balbale et al. (2016) on veterans with multiple chronic conditions [16, 27, 51]. In addition, participants with higher educational levels reported lower levels of care coordination. However, the opposite was found by Hawley et al. (2010) in a study of (ethnically) diverse patients with breast cancer [43]. It is possible that the correlation between lower educational levels and higher perceived care coordination is an effect of expectations and knowledge [16, 51]. Patients who know more about the healthcare system, treatments, and their rights may have more demands than those with less knowledge. Thus, knowledge and perceptions about care coordination may lead to a more critical view of their own situation.

Although the association between disease frequency and care coordination has been described numerous times in the literature, the expected difference in perceived care coordination was not observed in our analysis [7–11]. Social support groups may mediate the absence of this connection. However, there was a correlation between social support and patient activity [38, 52]. The higher activation of patients in social support groups may also mitigate coordination-related issues. Hence, in further investigations, the factor of being in a social support group should be actively surveyed and checked in the calculations.

Interestingly, we found a significant correlation of the treatment location and the total score of the CCI German version as well as the dimension “need-based navigation.” Patients who were treated in a hospital had a score of up to 10 points higher than patients who were treated by a private practice oncologist. These correlations were of major interest because a fixed contact person was mentioned in interviews with patients and physicians as an aspect of good coordination. This fixed-contact person is more likely to be guaranteed in the setting of treatment with a registered oncologist than in a large hospital with different physicians working different shifts. The significance of the treatment location becomes particularly evident in patients who are treated by a private practice urologist versus a hospital treatment. Although male patients reported a higher (better) care coordination, those treated by a private practice urologist had lower CCI scores. However, this correlation was not statistically significant. In dimension 2 (“need-based navigation”), the difference was statistically significant. Thus, male patients who are treated by a private practice urologist experience lower care coordination in general, especially for need-based navigation, than males treated in a hospital. This is meaningful because urologists play an important role in the healthcare of men [53–55]. They are often the initial points of contact for men in the healthcare system. Male patients with urological cancer rely mainly on urologists as sources of information [55]. Navigation and care coordination may be easier in hospitals. Perhaps the distances are shorter, and there are in-house diagnostic and treatment opportunities and regular communication and treatment procedures. Okado et al. (2020a) also found a significant correlation between practice settings and navigation scores in multivariable analysis [2]. Patients treated in hospitals felt that they were better guided than those treated in private practices. This finding was consistent with the results of the original instrument.

A surprising finding was the clarity and direction of the association between sex and perceived care coordination. The association was strong for both dimensions and total score. Male cancer patients perceived higher care coordination, higher need-based navigation, and better communication with their attending physicians. There is no clear consensus on this finding. According to Ayanian et al. (2005), male patients had a minimally lower coordination score than female patients [27]. Okado et al. (2020a) showed a clear difference in care coordination scores for patients’ sex in a group of low-coordination patients [2]. Other studies did not mention the effect of gender on care coordination [3, 28, 56]. However, none have focused on gender differences in care coordination perception. The lower score for women was initially surprising. Women use preventive services, visit doctors, and seek cancer-related information more often [54, 57, 58]. At this point, one might expect the healthcare system to adapt well to the needs of women. However, women may be more critical. They assessed their health condition as worse than men [57, 59]. This difference in health assessments decreased the older the participants and the higher their education level. The reason for the sex difference in health assessment is assumed to be the difference in awareness of physical and psychological changes [57, 60–65]. Male and older patients are more likely to take a passive or collaborative role in treatment decision-making than female or younger patients, who prefer an active role in shared decision-making [66, 67]. Weiner et al. (2020) noted that male healthcare needs are more focused on healing and female healthcare needs are more focused on prevention [54]. A 1999 review [68] clearly demonstrated the different needs and strategies of female patients in terms of communication, satisfaction, and effectiveness of medical encounters. Men provided fewer explanations, reported fewer issues, and solved problems individually. Women responded more to these questions than men did. Communicative effort was higher in female patients [68]. However, sex also affects the perceptions of communication partners during medical consultations [68, 69]. Thus, women and younger patients may have higher expectations regarding communication and treatment processes. In our sample, female patients were younger than male patients.

According to these findings, further investigations on care coordination should address sex differences and the roles and expectations of patients in care coordination. In addition, since this validation only included a German population, adaptation and further validation for other German speaking countries are needed.

### Strengths and Limitations

Our study has several strengths. The topic of care coordination for patients with cancer was addressed using a multi-method approach. We also explored this topic from the perspectives of physicians and patients. We recruited a sufficiently large sample. The German version of the CCI has excellent internal consistency and good face and content validity. Therefore, the CCI German version was the first valid instrument to measure cancer patients’ perceptions of care coordination in the German language.

Our study had some limitations. (I) The first translation of the CCI was conducted by a master’s student (female) and not by a professional translator or native English speaker. However, the first translation was from English to German, and the master’s student had experience in the use of English-language, health science texts. (II) We did not have any information on the educational qualifications of the interviewed patients. (III) The interviewer (AW) knew two of the participants before the interview. It is possible that this acquaintance influenced the interviews. (IV) We piloted the instrument only in online interviews. Validation of the CCI German version was mainly based on an online survey. Thus, patients without internet access or who had illness-related limitations in using the internet could not participate. (V) Inductive coding of the interviews was not conducted independently or in parallel by the two raters. One rater (AW) coded the interviews, and the other (JL) reviewed these ratings. A categorical system was developed through collaboration. (VI) We did not define rare cancer. There are some cut-offs to define the rarity of a cancer, but we did not specify this for our sample recruitment. We trusted the participants’ personal details and stated that their cancer was rare. However, the rarity of this condition remains unknown. (VII) We did not ask whether the participants were active in the support group. Support groups can play a navigational role, provide support for coordination, and mitigate issues related to care coordination [6, 70, 71]. Our recruitment strategy suggested a high number of participants in support groups. (VIII) Our sample was younger than the average cancer patients in Germany at the time of illness, which is why our sample is not representative of German cancer patients. (IX) We focused on patients with the most common and rarest cancers. This preselection can bias the CCI validation results. However, this strategy made it possible to include the spectrum of care. For the most common cancers in Germany, breast cancer and prostate cancer, there have been treatment guidelines for the highest quality level (S3) since 2004/2009 and patient guidelines to support coordination and navigation during the treatment process [55]. For recruited patients with rare cancers, there are only guidelines of lower quality (S2k—consent-based, not evidence-based) and no patient guidelines. We conclude that the lack of evidence-based and patient guidelines is reflected in the very different treatment paths. (X) The high values of Cronbach’s alpha could be an indication of redundant questions in the questionnaire.

## Conclusion

The CCI German version is a valid instrument for measuring cancer patient perceptions of care coordination in the German language. The two domains reflect important aspects of care. Further research should address care coordination for rare and common cancers as well as differences in perceptions of care coordination between men and women. Surveys with larger sample sizes and different types of cancer are desirable.

## Supplementary Information

Supplementary Material 1.

Supplementary Material 2.

Supplementary Material 3.

Supplementary Material 4.

## Data Availability

The datasets supporting the conclusions of this study are available upon request from the corresponding author.
