# Supplementary material for: Translation, adaptation, and validation of the Care Coordination Instrument for cancer patients
Source: BMC Health Serv Res. 2025 Jan 3;25:13. doi: 10.1186/s12913-024-12123-4 (PMC11697633; doi:10.1186/s12913-024-12123-4)
Supplement: Supplementary file 1 — Supplementary Material 1. [file 12913_2024_12123_MOESM1_ESM.docx]

**Interview guide**

***Initial questions***

What does good coordination of medical care mean to you? What do you associate with it? What terms do you associate with it?

***Presentation of the CCI-German version: discuss the -German version step by step, starting with the introduction, addressing each item individually.***

Please read through the questionnaire and let me know your thoughts as you read. What impression do you have? What might not be understandable? We start with the introduction.

***Question on special items or wordings. e.g.:***

From your point of view, is that easy to answer for a patient? What do you think is meant here by "coordinating my cancer treatment", what is included? What do you think is meant here by "financial aspects"?

***Final questions after finishing the questionnaire***

Please look at the individual items again to see whether they cover your idea of good care coordination. Do you have any comments on individual items?

In the original instrument, the items usually asked about the oncologist. We suggest asking about the doctor in charge of the patient. How do you see this?

Which items do you think are problematic? Was there an item that you had to think about longer?
