# Supplementary material for: Translation, adaptation, and validation of the Care Coordination Instrument for cancer patients
Source: BMC Health Serv Res. 2025 Jan 3;25:13. doi: 10.1186/s12913-024-12123-4 (PMC11697633; doi:10.1186/s12913-024-12123-4)
Supplement: Supplementary file 2 — Supplementary Material 2. [file 12913_2024_12123_MOESM2_ESM.docx]

**Translation and statistics for each item**

| No. | Original | Final German version | item-total correlation | Mean (SD) |
| --- | --- | --- | --- | --- |
| 1 | It was easy to schedule visits with my primary cancer doctor. | Es war einfach, Termine mit meiner Ärztin/meinem Arzt zu vereinbaren. | 0.62 | 1.97 (0.82) |
| 2 | I was provided information or received assistance for any emotional, financial, or social issues that might be of concern to me. | Ich erhielt Informationen oder Unterstützung auch für nicht-medizinische Fragen, die für mich von Bedeutung sind (z.B. emotionale, finanzielle oder soziale Fragen). | 0.53 | 1.26 (0.87) |
| 3 | In general, all procedures were explained to me. | Im Großen und Ganzen wurden mir alle Abläufe (z.B. anstehender Untersuchungen, Chemotherapien oder anderer Behandlungen) erklärt. | 0.57 | 1.91 (0.85) |
| 4 | Sometimes I have duplicate tests. | Manchmal werden bei mir Untersuchungen doppelt durchgeführt. (Gemeint sind NICHT reguläre Verlaufskontrollen.) | 0.20 | 2.18 (0.76) |
| 5 | My cancer doctor always reviews my past and current medical history with me. | Meine Ärztin/mein Arzt bespricht mit mir üblicherweise den Verlauf meiner Erkrankung seit dem letzten Besuch und den aktuellen Stand. | 0.65 | 1.97 (0.80) |
| 6 | My cancer doctor explains different treatment options to me. | Meine Ärztin/mein Arzt erklärt mir genau verschiedene Behandlungsmöglichkeiten. | 0.68 | 1.68 (0.84) |
| 7 | It is clearly explained to me what the role of doctors from different specialties is for my care. | Es wird mir verständlich erklärt, welche Rolle die Ärztinnen und Ärzte der verschiedenen Fachrichtungen für meine Versorgung haben. | 0.69 | 1.45 (0.87) |
| 8 | I feel that if I need support groups for my emotional wellbeing or for social concerns, my doctor or a member of his/her team would provide that information to me. | Wenn ich emotionalen Unterstützungsbedarf habe, bespricht meine Ärztin/mein Arzt oder ein Mitglied des Teams verschiedene Unterstützungsangebote mit mir (z.B. Selbsthilfegruppen, Gesundheitsapps, Beratung der Krebsgesellschaften). | 0.61 | 1.10 (0.85) |
| 9 | I was informed of financial aspects of cancer care. | Finanzielle Aspekte der Krebserkrankung (z.B. Verdienstausfälle, mögliche Zusatzkosten für Behandlung und Diagnostik) wurden mit mir besprochen. | 0.47 | 0.81 (0.82) |
| 10 | I have a good understanding of my treatment plan. | Ich habe einen guten Überblick über meinen Behandlungsplan. | 0.66 | 1.95 (0.72) |
| 11 | My future appointments are easy to make. | Meine anstehenden Termine sind einfach zu vereinbaren. | 0.53 | 1.90 (0.75) |
| 12 | I feel that my cancer doctor communicates frequently with other doctors involved in my care. | Zwischen den Ärztinnen und Ärzten, die an meiner Behandlung beteiligt sind, besteht ein Informationsaustausch. | 0.66 | 1.45 (0.88) |
| 13 | I know which of my doctors to call if I have questions or any complications from my treatments. | Ich weiß, welche meiner Ärztinnen oder Ärzte ich kontaktieren muss, wenn ich Fragen habe oder es zu Komplikationen bei meinen Behandlungen kommt. | 0.63 | 1.90 (0.84) |
| 14 | When I call my cancer doctor I receive a return call in a timely fashion. | Wenn ich meine Ärztin/meinen Arzt anrufe, erhalte ich zeitnah einen Rückruf. | 0.64 | 2.73 (0.84) |
| 15 | My cancer doctor had all the information they s/he needed, such as test results, to make decisions about my treatment. | Meine Ärztin/mein Arzt hat alle notwendigen Informationen, wie z. B. Untersuchungsergebnisse, die in die Entscheidungen zu meiner Behandlung einfließen. | 0.57 | 2.15 (0.72) |
| 16 | My cancer doctor doesn’t know how much emotional support I need. | Meine Ärztin/mein Arzt kann nicht einschätzen, ob ich emotionale Unterstützung brauche. | 0.57 | 1.10 (0.75) |
| 17 | Someone from my doctor’s office reaches out and contacts me after visits to check whether I have any problems or concerns. | Initial translated: Jemand aus dem Behandlungsteam meldet sich nach meinen Besuchen bei mir, um nachzufragen, ob ich irgendwelche Probleme oder Bedenken habe. |  |  |
|  |  | final (alternative) Item: Wenn etwas offengeblieben sein sollte, meldet sich jemand aus dem Behandlungsteam nach meinen Besuchen bei mir, um diese Frage zu klären. | 0.64 | 1.10 (0.87) |
| 18 | I feel like my cancer doctor or staff would answer all my questions about my treatment. | Meine Ärztin/mein Arzt oder ihre/seine Mitarbeitenden beantworten alle meine Fragen zu meiner Behandlung. | 0.55 | 1.93 (0.74) |
| 19 | My cancer doctor recommended that I get a second opinion. | Meine Ärztin/mein Arzt hat mir das Angebot gemacht, eine zweite Meinung einzuholen. | 0.37 | 1.17 (0.99) |
| 20 | I feel that my cancer doctor would make it easy to get a referral with any other specialists I might need to see. | Meine Ärztin/mein Arzt macht es mir leicht, eine Überweisung zu anderen Spezialistinnen oder Spezialisten zu bekommen. | 0.60 | 2.02 (0.90) |
| 21 | I feel like my cancer doctor thinks about my family or living situation when planning my treatments. | Ich denke, dass meine Ärztin/mein Arzt bei der Planung meiner Behandlungen meine Lebenssituation oder meine Familie im Blick hat. | 0.59 | 1.20 (0.88) |
| 22 | If I had pain, discomfort or any other symptoms my cancer doctor would do everything s/he could to control this. | Wenn Schmerzen, Unwohlsein oder andere Symptome auftreten, wird meine Ärztin/mein Arzt alles tun, um dies in den Griff zu bekommen. | 0.72 | 1.82 (0.84) |
| 23 | If I had a serious symptom at home, the first thing I do is to go to the Emergency Room. | Initial translated: Wenn ich zu Hause ein ernstzunehmendes Symptom hätte, würde ich als erstes in die Notaufnahme gehen“* |  |  |
|  |  | final (alternative) Item: Ich habe Informationen bekommen, an welche Personen / Institutionen ich mich wenden kann, wenn zu Hause ein ernstzunehmendes Symptom auftritt.** | 0.56 | 1.18 (0.89) |
| 24 | I feel like my cancer doctor spends enough time with me. | Ich habe das Gefühl, dass sich meine Ärztin/mein Arzt genug Zeit für mich nimmt. | 0.67 | 1.71 (0.87) |
| 25 | I have trouble scheduling an appointment at the time and date that is good for me. | Ich habe Schwierigkeiten, Termine zu einer für mich passenden Zeit und einem passenden Datum zu vereinbaren. | 0.50 | 1.88 (0.80) |
| 26 | I have a family member, a close relative or a friend who helped coordinate my cancer care. | Mich hat ein Familienmitglied, eine Freundin oder ein Freund unterstützt, meine Krebsbehandlung zu koordinieren. | 0.05 | 1.37 (1.06) |
| 27 | I felt like my care was impacted by the type of insurance I have. | Ich hatte den Eindruck, dass meine Versorgung durch die Art meiner Versicherung negativ beeinflusst wurde. | 0.38 | 1.91 (0.90) |
| 28 | My cancer doctor suggested that I consider a clinical trial. | Meine Ärztin/mein Arzt hat vorgeschlagen, über die Teilnahme an einer klinischen Studie nachzudenken. | 0.37 | - 1. (0.97) |
| 29 | I feel that my cancer care is well-coordinated. | Ich habe das Gefühl, dass meine Krebsversorgung zwischen allen Beteiligten gut abgestimmt ist. | 0.73 | 1.47 (0.84) |

* Follow-up Questions: „Ich habe beim Lesen der Frage gedacht: Das Symptom ist so schwerwiegend, dass mir nur in der Notaufnahme geholfen werden kann. „(ja, eher ja, eher nein, nein); „Alle anderen Möglichkeiten, z.B. Anruf der Notfallnummer, wurden schon versucht.“ (ja, eher ja, eher nein, nein); „Das Aufsuchen der Notaufnahme wurde mir für so einen Fall von meiner Ärztin/meinem Arzt empfohlen.“ (ja, eher ja, eher nein, nein); „Ich weiß nicht, an wen ich mich sonst wenden soll.“ (ja, eher ja, eher nein, nein) / “When I read the question, I thought: The symptom is so severe that I can only be helped in the emergency room. “ (yes, rather yes, rather no, no); “All other options, e.g. calling the emergency number, have already been tried.” (yes, rather yes, rather no, no); “Going to the emergency room was recommended to me by my doctor for such a case.” (yes, rather yes, rather no, no); “I don't know who else to turn to.” (yes, rather yes, rather no, no)

** Follow-up Question: „Sie haben der Aussage in Frage 26 (voll und ganz) zugestimmt. An welche Person/Institution haben sie gedacht? (Freitext)“ / “You (fully) agreed with the statement in question 26. Which person/institution did you have in mind? (free text)”
