# Supplementary material for: Translation, adaptation, and validation of the Care Coordination Instrument for cancer patients
Source: BMC Health Serv Res. 2025 Jan 3;25:13. doi: 10.1186/s12913-024-12123-4 (PMC11697633; doi:10.1186/s12913-024-12123-4)
Supplement: Supplementary file 3 — Supplementary Material 3. [file 12913_2024_12123_MOESM3_ESM.docx]

Care Coordination Instrument German Version - Scoring Guide

***Kodierung***

Antworten auf Items 1, 2, 3, 5, 6, 7, 8, 9, 10, 11, 12, 13, 14, 15, 17, 18, 19, 20, 21, 22, 23, 25, 27, 28, 29 werden wie folgt kodiert:

| Stimme gar nicht zu = 0 | Stimme nicht zu = 1 | Stimme zu = 2 | Stimme voll und ganz zu = 3 |
| --- | --- | --- | --- |

Antworten auf Items 4, 16, 24, 26, werden *reverse* kodiert

| Stimme gar nicht zu = 3 | Stimme nicht zu = 2 | Stimme zu = 1 | Stimme voll und ganz zu = 0 |
| --- | --- | --- | --- |

***Berechnung des total scores und der einzelnen domains***

*Total Score*
Der *total score* bildet sich aus der Summe aller Items.

Ergebnisse der Validierungsstudie:

Mittelwert Total score: 47.16 (SD 14.25)

Interne Konsistenz: Cronbach’s α = .931

*Domain 1 – Kommunikation und Information*
Summe der Items: 1, 3, 5, 6, 10, 11, 12, 13, 14, 15, 17, 19, 21, 23, 24, 29.

Ergebnisse der Validierungsstudie:

Mittelwert Domain 1: 30.14 (SD 8.93)

Interne Konsistenz: Cronbach’s α =.924

*Domain 2 – Bedarfsbasierte interprofessionelle Navigation*

Summe der Items: 2, 3, 4, 6, 7, 8, 9, 11, 12, 16, 18, 20, 22, 25, 26, 27, 28.

Ergebnisse der Validierungsstudie:

Mittelwert Domain 2: 23.99 (SD 8.37)

Interne Konsistenz: Cronbach’s α =.868

Fragebogen zur Versorgungskoordination für Krebspatientinnen und -patienten

Die folgenden Fragen beziehen sich auf Ihre Erfahrungen und Einschätzungen der Versorgungskoordination als Krebspatientin oder Krebspatient. Versorgungskoordination bezieht sich darauf, wie die Versorgung Ihrer Krebserkrankung zwischen Ihnen und den an Ihrer Versorgung beteiligten Gesundheitsdienstleistern (z.B. Kliniken, fachärztliche Praxen, Pflegedienste oder Rehabilitationseinrichtungen) organisiert ist. Die Koordinierung der Versorgung umfasst Dinge wie die ...

- Kommunikation zwischen Ihnen und den Gesundheitsdienstleistern,
- Einfachheit der Vereinbarung und Verfügbarkeit von Terminen,
- Erreichbarkeit der zuständigen Ärztinnen und Ärzte
- Kommunikation zwischen Ihrer betreuenden Ärztin bzw. Ihrem betreuenden Arzt und anderen Gesundheitsdienstleistern.

Ihre betreuende Ärztin bzw. Ihr betreuender Arzt ist maßgeblich für die Behandlung Ihrer Krebserkrankung verantwortlich. Das kann zum Beispiel Ihr Onkologe, Ihre Hausärztin oder auch Gynäkologin bzw. Urologe sein. Im Folgenden wird „meine Ärztin/mein Arzt“ verwendet. Bitte denken Sie hierbei immer an Ihre betreuende Ärztin bzw. Ihren betreuenden Arzt.

Die Fragen können den gesamten Behandlungszyklus umfassen – Diagnostik, Behandlung, Rehabilitation, Nachsorge. Je nachdem an welchem Punkt Ihrer Behandlung Sie sich befinden, sind ihre bisherigen Erfahrungen und aktuellen Einschätzungen erfragt.

Bitte kreuzen Sie für jede Frage die Antwort an, die für Sie am besten zutrifft. Versuchen Sie nach Möglichkeit, für jede Frage eine Antwort auszuwählen; es gibt keine richtige oder falsche Antwort. Wenn Sie mehr als eine Behandlung für Ihre Krebserkrankung hatten, wählen Sie bitte die Antwort aus, die am häufigsten für Sie zutraf.

|  | Stimme  gar nicht zu | Stimme  nicht zu | Stimme zu | Stimme voll und ganz zu |
| --- | --- | --- | --- | --- |
| 1. Es war einfach, Termine mit meiner Ärztin/meinem Arzt zu vereinbaren. | ☐ | ☐ | ☐ | ☐ |
| 1. Ich erhielt Informationen oder Unterstützung auch für nicht-medizinischen Fragen, die für mich von Bedeutung sind (z.B. emotionale, finanzielle oder soziale Fragen). | ☐ | ☐ | ☐ | ☐ |
| 1. Im Großen und Ganzen wurden mir alle Abläufe (z.B. anstehender Untersuchungen, Chemotherapien oder anderer Behandlungen) erklärt. | ☐ | ☐ | ☐ | ☐ |
| 1. Manchmal werden bei mir Untersuchungen doppelt durchgeführt.  (Gemeint sind NICHT reguläre Verlaufskontrollen) | ☐ | ☐ | ☐ | ☐ |
| 1. Meine Ärztin/mein Arzt bespricht mit mir üblicherweise den Verlauf meiner Erkrankung seit dem letzten Besuch und den aktuellen Stand. | ☐ | ☐ | ☐ | ☐ |
| 1. Meine Ärztin/mein Arzt erklärt mir genau verschiedene Behandlungsmöglichkeiten. | ☐ | ☐ | ☐ | ☐ |
| 1. Es wird mir verständlich erklärt, welche Rolle die Ärztinnen und Ärzte der verschiedenen Fachrichtungen für meine Versorgung haben. | ☐ | ☐ | ☐ | ☐ |
| 1. Wenn ich emotionalen Unterstützungsbedarf habe, bespricht meine Ärztin/mein Arzt oder ein Mitglied des Teams verschiedene Unterstützungsangebote mit mir (z.B. Selbsthilfegruppen, Gesundheitsapps, Beratung der Krebsgesellschaften). | ☐ | ☐ | ☐ | ☐ |
| 1. Finanzielle Aspekte der Krebserkrankung (z.B. Verdienstausfälle, mögliche Zusatzkosten für Behandlung und Diagnostik) wurden mit mir besprochen. | ☐ | ☐ | ☐ | ☐ |
| 1. Ich habe einen guten Überblick über meinen Behandlungsplan. | ☐ | ☐ | ☐ | ☐ |
| 1. Meine anstehenden Termine sind einfach zu vereinbaren. | ☐ | ☐ | ☐ | ☐ |
| 1. Zwischen den Ärztinnen und Ärzten, die an meiner Behandlung beteiligt sind, besteht ein Informationsaustausch. | ☐ | ☐ | ☐ | ☐ |
| 1. Ich weiß, welche meiner Ärztinnen oder Ärzte ich kontaktieren muss, wenn ich Fragen habe oder es zu Komplikationen bei meinen Behandlungen kommt. | ☐ | ☐ | ☐ | ☐ |
| 1. Wenn ich meine Ärztin/meinen Arzt anrufe, erhalte ich zeitnah einen Rückruf. | ☐ | ☐ | ☐ | ☐ |
| 1. Meine Ärztin/mein Arzt hat alle notwendigen Informationen, wie z. B. Untersuchungsergebnisse, die in die Entscheidungen zu meiner Behandlung einfließen. | ☐ | ☐ | ☐ | ☐ |
| 1. Meine Ärztin/mein Arzt kann **nicht** einschätzen, ob ich emotionale Unterstützung brauche. | ☐ | ☐ | ☐ | ☐ |
| 1. Meine Ärztin/mein Arzt oder ihre/seine Mitarbeitenden beantworten alle meine Fragen zu meiner Behandlung. | ☐ | ☐ | ☐ | ☐ |
| 1. Meine Ärztin/mein Arzt hat mir das Angebot gemacht, eine zweite Meinung einzuholen. | ☐ | ☐ | ☐ | ☐ |
| 1. Meine Ärztin/mein Arzt macht es mir leicht, eine Überweisung zu anderen Spezialistinnen oder Spezialisten zu bekommen. | ☐ | ☐ | ☐ | ☐ |
| 1. Ich denke, dass meine Ärztin/mein Arzt bei der Planung meiner Behandlungen meine Lebenssituation oder meine Familie im Blick hat. | ☐ | ☐ | ☐ | ☐ |
| 1. Wenn Schmerzen, Unwohlsein oder andere Symptome auftreten, wird meine Ärztin/mein Arzt alles tun, um dies in den Griff zu bekommen. | ☐ | ☐ | ☐ | ☐ |
| 1. Ich habe Informationen bekommen, an welche Personen / Institutionen ich mich wenden kann, wenn zu Hause ein ernstzunehmendes Symptom auftritt. | ☐ | ☐ | ☐ | ☐ |
| 1. Ich habe das Gefühl, dass sich meine Ärztin/mein Arzt genug Zeit für mich nimmt. | ☐ | ☐ | ☐ | ☐ |
| 1. Ich habe Schwierigkeiten, Termine zu einer für mich passenden Zeit und einem passenden Datum zu vereinbaren. | ☐ | ☐ | ☐ | ☐ |
| 1. Mich hat ein Familienmitglied, eine Freundin oder ein Freund unterstützt, meine Krebsbehandlung zu koordinieren. | ☐ | ☐ | ☐ | ☐ |
| 1. Ich hatte den Eindruck, dass meine Versorgung durch die Art meiner Versicherung negativ beeinflusst wurde. | ☐ | ☐ | ☐ | ☐ |
| 1. Meine Ärztin/mein Arzt hat vorgeschlagen, über die Teilnahme an einer klinischen Studie nachzudenken. | ☐ | ☐ | ☐ | ☐ |
| 1. Ich habe das Gefühl, dass meine Krebsversorgung zwischen allen Beteiligten gut abgestimmt ist. | ☐ | ☐ | ☐ | ☐ |
| 1. Wenn etwas offen geblieben sein sollte, meldet sich jemand aus dem Behandlungsteam nach meinen Besuchen bei mir, um diese Frage zu klären. | ☐ | ☐ | ☐ | ☐ |

Vielen Dank, dass Sie diese Befragung teilgenommen haben.
