# Supplementary material for: Translation, adaptation, and validation of the Care Coordination Instrument for cancer patients
Source: BMC Health Serv Res. 2025 Jan 3;25:13. doi: 10.1186/s12913-024-12123-4 (PMC11697633; doi:10.1186/s12913-024-12123-4)
Supplement: Supplementary file 4 — Supplementary Material 4. [file 12913_2024_12123_MOESM4_ESM.docx]

**Group differences for each item**

|  | female | male |  |
| --- | --- | --- | --- |
|  | mean (SD) | mean (SD) | χ^2^ |
| Es war einfach, Termine mit meiner Ärztin/meinem Arzt zu vereinbaren. | 1.89 (0.79) | 2.11 (0.87) |  |
| Ich erhielt Informationen oder Unterstützung auch für nicht-medizinische Fragen, die für mich von Bedeutung sind  (z.B. emotionale, finanzielle oder soziale Fragen). | 1.17 (0.79) | 1.41 (0.97) | *p* = 0.037 |
| Im Großen und Ganzen wurden mir alle Abläufe (z.B. anstehender Untersuchungen, Chemotherapien oder anderer Behandlungen) erklärt. | 1.88 (0.85) | 1.97 (0.86) |  |
| Manchmal werden bei mir Untersuchungen doppelt durchgeführt. (Gemeint sind NICHT reguläre Verlaufskontrollen.) * | 2.28 (0.70) | 2.01 (0.83) |  |
| Meine Ärztin/mein Arzt bespricht mit mir üblicherweise den Verlauf meiner Erkrankung seit dem letzten Besuch und den aktuellen Stand. | 1.90 (0.76) | 2.11 (0.84) |  |
| Meine Ärztin/mein Arzt erklärt mir genau verschiedene Behandlungsmöglichkeiten. | 1.57 (0.81) | 1.86 (0.87) |  |
| Es wird mir verständlich erklärt, welche Rolle die Ärztinnen und Ärzte der verschiedenen Fachrichtungen für meine Versorgung haben. | 1.30 (0.86) | 1.68 (0.85) | *p* = 0.029 |
| Wenn ich emotionalen Unterstützungsbedarf habe, bespricht meine Ärztin/mein Arzt oder ein Mitglied des Teams verschiedene Unterstützungsangebote mit mir (z.B. Selbsthilfegruppen, Gesundheitsapps, Beratung der Krebsgesellschaften). | 0.98 (0.80) | 1.30 (0.90) | *p* = 0.044 |
| Finanzielle Aspekte der Krebserkrankung (z.B. Verdienstausfälle, mögliche Zusatzkosten für Behandlung und Diagnostik) wurden mit mir besprochen. | 0.74 (0.77) | 0.92 (0.89) |  |
| Ich habe einen guten Überblick über meinen Behandlungsplan. | 1.82 (0.71) | 2.18 (0.69) | *p* = 0.004 |
| Meine anstehenden Termine sind einfach zu vereinbaren. | 1.84 (0.72) | 1.99 (0.80) |  |
| Zwischen den Ärztinnen und Ärzten, die an meiner Behandlung beteiligt sind, besteht ein Informationsaustausch. | 1.39 (0.87) | 1.57 (0.89) |  |
| Ich weiß, welche meiner Ärztinnen oder Ärzte ich kontaktieren muss, wenn ich Fragen habe oder es zu Komplikationen bei meinen Behandlungen kommt. | 1.76 (0.84) | 2.12 (0.79) | *p* = 0.014 |
| Wenn ich meine Ärztin/meinen Arzt anrufe, erhalte ich zeitnah einen Rückruf. | 2.70 (0.83) | 2.80 (0.86) |  |
| Meine Ärztin/mein Arzt hat alle notwendigen Informationen, wie z. B. Untersuchungsergebnisse, die in die Entscheidungen zu meiner Behandlung einfließen. | 2.09 (0.71) | 2.27 (0.73) |  |
| Meine Ärztin/mein Arzt kann nicht einschätzen, ob ich emotionale Unterstützung brauche. * | 1.06 (0.70) | 1.18 (0.83) | *p* = 0.020 |
| Meine Ärztin/mein Arzt oder ihre/seine Mitarbeitenden beantworten alle meine Fragen zu meiner Behandlung. | 1.89 (0.71) | 2.03 (0.78) |  |
| Meine Ärztin/mein Arzt hat mir das Angebot gemacht, eine zweite Meinung einzuholen. | 1.01 (0.93) | 1.43 (1.04) | *p* = 0.017 |
| Meine Ärztin/mein Arzt macht es mir leicht, eine Überweisung zu anderen Spezialistinnen oder Spezialisten zu bekommen. | 1.95 (0.92) | 2.18 (0.82) |  |
| Ich denke, dass meine Ärztin/mein Arzt bei der Planung meiner Behandlungen meine Lebenssituation oder meine Familie im Blick hat. | 1.08 (0.84) | 1.42 (0.91) |  |
|  | female | male |  |
|  | mean (SD) | mean (SD) | χ^2^ |
| Wenn Schmerzen, Unwohlsein oder andere Symptome auftreten, wird meine Ärztin/mein Arzt alles tun, um dies in den Griff zu bekommen. | 1.78 (0.82) | 1.92 (0.85) |  |
| Ich habe Informationen bekommen, an welche Personen/ Institutionen ich mich wenden kann, wenn zu Hause ein ernstzunehmendes Symptom auftritt. | 1.10 (0.86) | 1.33 (0.93) |  |
| Ich habe das Gefühl, dass sich meine Ärztin/mein Arzt genug Zeit für mich nimmt. | 1.63 (0.91) | 1.86 (0.79) |  |
| Ich habe Schwierigkeiten, Termine zu einer für mich passenden Zeit und einem passenden Datum zu vereinbaren. * | 1.84 (0.78) | 1.95 (0.83) |  |
| Mich hat ein Familienmitglied, eine Freundin oder ein Freund unterstützt, meine Krebsbehandlung zu koordinieren. | 1.30 (1.04) | 1.46 (1.10) |  |
| Ich hatte den Eindruck, dass meine Versorgung durch die Art meiner Versicherung negativ beeinflusst wurde. * | 1.94 (0.90) | 1.86 (0.91) |  |
| Meine Ärztin/mein Arzt hat vorgeschlagen, über die Teilnahme an einer klinischen Studie nachzudenken. | 0.93 (0.93) | 1.15 (1.02) |  |
| Ich habe das Gefühl, dass meine Krebsversorgung zwischen allen Beteiligten gut abgestimmt ist. | 1.40 (0.80) | 1.59 (0.91) |  |
| Wenn etwas offengeblieben sein sollte, meldet sich jemand aus dem Behandlungsteam nach meinen Besuchen bei mir, um diese Frage zu klären. | 1.02 (0.83) | 1.22 (0.93) |  |

** reverse items;*
